# Experience of life quality from patients with aplastic anemia: a descriptive qualitative study

**DOI:** 10.1186/s13023-023-02993-y

**Published:** 2023-12-21

**Authors:** Ting Liu, Yue Pan, Menghua Ye, Qiuhua Sun, Xinghong Ding, Min Xu

**Affiliations:** 1 School of Basic Medical Sciences, Zhejiang Chinese Medical University, No.548 Binwen Road, Binjiang District, Hangzhou, Zhejiang Province China; 2 School of Nursing, Zhejiang Chinese Medical University, No.548 Binwen Road, Binjiang District, Hangzhou, Zhejiang Province China; 3 School of Nursing and Health, Zhejiang Changzheng Vocational and Technical College, No.525 Liuhe Road, Xihu District, Hangzhou, Zhejiang Province China; 4 The First Affiliated Hospital of Zhejiang Chinese Medical University (Zhejiang Provincial Hospital of Chinese Medcine), No.54 Youdian Road, Shangcheng District, Hangzhou, Zhejiang Province China

**Keywords:** Aplastic anemia, Experiences, Qualitative study, Quality of life

## Abstract

**Background:**

Despite the increasing incidence of aplastic anemia in China, few studies have explored its effect on the patients’ quality of life from the perspective of these patients. In fact, patients with aplastic disorder live with the disease for a long time, and need to face a variety of difficult realities, including multiple disease symptoms and drug side effects, heavy burden of medical costs, difficulties in social reintegration, and negative emotional distress. Therefore, this study used descriptive qualitative research to explore the direct and rich quality-of-life experiences of patients with aplastic anemia.

**Methods:**

A total of 19 patients with aplastic anemia were recruited in this study using purposive sampling combined with maximum variation strategy. 5 of the patients with AA were from northern China, and the others were from southern China. Data were collected using semi-structured interviews and analyzed using the conventional content analysis method.

**Results:**

This study yielded important information about the experiences of patients with aplastic anemia in China. The content analysis method finally identified 3 themes and 9 sub-themes, including: physical symptoms (declining physical capacity, treatment-related symptoms, changes in body image), psychological symptoms (mood changes related to the stage of the disease, change in self-image, growth resulting from the disease experience), social burden (decline in career development, perceived burden to the family, social stigma). Patients with AA from different regions didn’t show much difference in quality of life.

**Conclusions:**

Aplastic anemia affects the physical, psychological, and social aspects of patients’ lives. Therefore, health care providers need to consider the patients’ physical response and psychological feelings to provide relevant medical guidance and multi-channel social support that would improve their confidence and quality of life.

**Clinical trial registration:**

Name: Development and preliminary application of Quality of Life Scale for Patients with Aplastic Anemia. Number: ChiCTR2100047575. URL: http://www.chictr.org.cn/login.aspx?referurl=%2flistbycreater.aspx.

**Supplementary Information:**

The online version contains supplementary material available at 10.1186/s13023-023-02993-y.

## Background

Aplastic anemia (AA) is a bone marrow hematopoietic failure syndrome. It is characterized by pancytopenia and bone marrow cell reduction. It clinically manifests as anemia, bleeding, and infection [1]. Although the pathogenesis of AA has not yet been fully elucidated, immune abnormalities, especially abnormal activation of T lymphocytes and hyperfunction of the bone marrow damage, play a major role in its development [2]. The incidence of AA in East Asia is 2–3 times higher than in western countries, where it is about 2–3 cases per million people [3]. In China, the incidence of AA is about 7.4 cases per million people [4]. With the intensification of environmental pollution in recent years, the incidence of AA has shown an increasing trend [5]. Therefore, more clinical attention and research investment are needed to reduce environmental risk factors of AA.

The established treatment modalities for AA encompass immunosuppressive therapy (IST), hematopoietic stem cell transplantation (HSCT), and androgen-based hematopoietic therapy. Additionally, newer drugs like eltrombopag (Promacta®) and recombinant human thrombopoietin (Tebiao®) have gained approval for managing refractory AA [6]. According to Mikhaylova et al., the probability of a 10-year overall survival (OS) rate of patients with AA treated with IST was 90% [7]. In addition, a retrospective analysis of patients receiving HSCT showed a 5-year OS of 53% in patients over the age of 40 years, compared with 82% and 72% in patients under 20 years and aged 20–40 years, respectively [8]. With the development of new drugs, HSCT technology, and advances in the IST program, the survival time of patients with the disease has been greatly extended. However, AA and its treatment are often accompanied by oral ulcers, nausea and vomiting, diarrhea, decreased physical function, fatigue, anxiety, dry mouth, rash and respiratory problems, and risks of AA recurrence, secondary clonal disease, infection, graft-versus-host disease (GVHD), and so on [9, 10]. Regarding disease course, AA is characterized by a long treatment cycle and repeated disease changes. A previous study showed that 86% of patients with Fanconi anemia experienced psychiatric symptoms of anxiety/depression [11]. In severe cases, patients with AA may give up treatment and even commit suicide. Therefore, the various clinical manifestations of the disease and adverse reactions caused by drugs not only affect the patients’ physiological conditions but also negatively stimulate their mental health, lifestyle, and social role, significantly reducing the overall quality of life of patients with AA. For patients with AA living with chronic disease, the purpose of treatment is not only to prolong the survival period or improve clinical outcomes but also to improve their quality of life to achieve comprehensive physical, psychological, spiritual, and social rehabilitation.

Quality of life is a multi-dimensional, dynamic and subjective concept shaped by an individual physical health, mental state, social support and environmental factors [12]. Compared with traditional OS indicators, the quality of life can more comprehensively and accurately reflect the survival status of patients. Therefore, it has become an international prognostic measure of survival in patients. In recent years, there have been several reports on hematological diseases, mainly malignant hematological diseases, such as acute leukemia and multiple myeloma, but few studies have investigated non-malignant hematological diseases, especially AA. A survey of Chinese patients with AA shows that their quality of life is significantly decreased, and the more severe the anemia or the lower the platelet count, the lower the quality of life [13, 14]. In addition, aplastic anemia has the characteristics of long treatment cycle and repeated disease changes, resulting in about 52% of patients with AA with depression [15], and even lead to serious consequences such as suicide [24]. Foreign scholars have also paid attention to the reality that the quality of life of patients with AA is not optimistic. Professor Gorth and his team [16, 17] developed. Aplastic Anemia and/or Paroxysmal Nocturnal Hemoglobinuria-specific Quality of Life Questionnaire (QLQ-AA/PNH), it shows good internal consistency. Weisshaar et al. identified 11 symptom groups related to quality of life in AA/PNH patients and developed a questionnaire [18].

To sum up, the quality of life of patients with AA is a research topic that needs attention but lacks research investment. Fortunately, more and more scholars in the world have begun to pay attention to the quality of life of patients with AA and have begun to develop relevant assessment tools and relevant surveys. However, there is still a lack of in-depth research and extensive investigation. Moreover, due to differences in race, epidemiology, disease characteristics and medical conditions, the quality of life of Chinese patients with AA still needs to be further explored. Therefore, this study aimed to explore the quality of life experiences of patients with AA and to understand how AA influences the physical, psychological and social aspects of patients’ lives. We anticipate that the findings of this study will enhance the understanding of this group of patients by both health care providers and the general public. This will provide a basis for development of appropriate clinical interventions and management plans for this population and elicit support from society.

## Methods

### Design

A descriptive qualitative design was adopted in this study [19]. In this approach, researchers avoid using preconceived categories and instead immerse themselves in the data to uncover new insights into the phenomenon [19]. The study was reported using the COREQ criteria for reporting qualitative research [20].

### Setting and sample

Purposive sampling combined with maximum variation strategy [21] reflecting gender, age, course of disease, and treatments received since disease onset was used to select patients with AA from hematology wards of two hospital in China from April 2021 to October 2023. Eligible participants showed the following characteristics: (1) diagnosed with AA based on the Chinese criteria released in 2018 [22]; (2) older than 18 years; (3) knowledge of their diagnosis and progression; and (4) volunteered to participate in the study and had no communication barriers. Participants were excluded if they (1) had serious changes in heart, lung, liver, or kidney function that affect normal communication; (2) had a history of mental disorders; or (3) refused to interview on tape.

### Data collection

Data were collected using semi-structured interviews that were generally conducted face-to-face. Initially, an interview guide was developed based on a literature review focusing on the following seven questions: (1) How were you diagnosed with this disease? How did you feel when you were first diagnosed? (2) What medical treatments have you had in the past? Are there any side effects from these treatments? (3) How has your life (physical/mental/social/spiritual) changed since your illness? (4) How have these changes affected your quality of life? (5) What kind of help and support (hospital/family/community) have you received since your illness? (6) Are you satisfied with your current quality of life (physical/mental/social functioning/spiritual status)? (7) Can you describe your current life situation in 1–3 words? Interviews were conducted in private and quiet places chosen by the participants and were recorded. Firstly, the researcher introduced herself and engaged the participants in casual chats to create a relaxing atmosphere. Comprehensive field notes were meticulously recorded, encompassing observations, interactions, environmental context, and non-verbal cues exhibited by the participants. The researcher maintained a neutral stance, refraining from passing any subjective judgments on the interview data. On average, the interviews extended for approximately 45 min to an hour, and all sessions were audio-recorded with explicit consent from the patients.

### Data analysis

Upon completion, each audio-recorded interview was transcribed verbatim. All data were analyzed concurrently with data collection using the conventional content analysis method [19]. The first step involved reading the entire transcript of the interview several times to understand the participant’s experiences. Then, any narrative data that related to the patients with AA’ experiences of the quality of life were hand-coded line-by-line. Subsequently, the codes and concepts generated were grouped into subthemes and themes based on their similarities and differences. Finally, a definition for each theme and subtheme was created and supported with quotes from the data. Data collection and analysis procedures were conducted repeatedly until saturation was reached, with no further themes or subthemes emerging [23].

### Ethical considerations

Ethical approval for this study was obtained from the ethics committee of the First Affiliated Hospital of Zhejiang Chinese Medicine University with reference number 2021-K-213-01. Written informed consent was obtained from each participant. All participants were informed about the purposes of this study. They were also informed of their right to withdraw from the study at any time without a risk of any negative impacts on the services delivered to them. We also guaranteed the confidentiality of their personal information.

## Results

A total of 19 patients with AA were recruited for this study. 5 of the patients were from northern China and the other 14 were from southern China, and patients from different regions didn’t show much difference in quality of life. 8 patients were male and 11 were female, ranging in age from 19 to 67 years. Detailed demographic and clinical information is shown in Supplementary Table 1. The analysis yielded 3 themes and 9 subthemes (Supplementary Table 2; Supplementary Fig. 1).

### Theme 1. Physical symptoms

#### Declining physical capacity

Declining physical capacity refers to weakened physiological functions such as impaired organ function, reduced immune function, and decreased vitality of patients with AA. In this study, all 19 interviewees mentioned that they felt weakened and easily fatigued after the illness.

*Walking about 10 m, I feel quite tired*. (P5)

*After I got sick, I got tired easily, and it was easy to pant, and then my activities were not as good as before.* (P6)

*After my (hemoglobin) dropped from 74 to 46, the most noticeable feeling was fatigue, well, just wanting to sleep every day, occasionally climbing stairs as if my heart is going to beat out of my chest.* (P14)

Patients with AA often experience fatigue, primarily stemming from anemia characterized by reduced red blood cell counts or low hemoglobin levels (HGB < 100 g/L). Moreover, when individuals exhibit diminished white blood cell counts (ANC < 1.5 × 10^9^/L), they may be susceptible to infections and fever. Additionally, a low platelet count (PLT < 50 × 10^9^/L) can lead to heightened susceptibility to bleeding, which may manifest as frequent gum and nosebleeds, skin bruising, or prolonged menstrual cycles.

*(Before transplantation) you often get fever and white blood cells (low), and you get infected all at once.* (P9)

*Later, teething blood (gum bleeding) still happened again, even every night, which had a great impact on my daily life.* (P7)

*I haven’t been able to participate in some sports since middle school (due to low platelets), and I had to apply for a waiver for P.E. on my midterm, but I actually enjoy playing volleyball… Also, I have a very long period, which is annoying.* (P14)

#### Treatment-related symptoms

Treatment-related symptoms refer to various complications in patients with AA after treatment with cyclosporine, androgen, ATG/ALG, and HSCT, which greatly affect the patient’s quality of life and hinder the patients’ daily nutritional supplementation, necessary activities, and physical comfort. Separately, patients with chronic AA are usually treated with cyclosporine (an immunosuppressant) in combination with androgens. The administration of cyclosporine may lead to gingival hyperplasia as well as various digestive discomforts like nausea, vomiting, reduced appetite, and altered taste perception. Androgens use can impact the female reproductive system, causing increased body hair, amenorrhea, clitoral enlargement, deepening of the voice, and breast reduction. When administered during childhood, androgens can accelerate growth and bone maturation, potentially leading to premature epiphyseal fusion.

*The teeth in the mouth grow fleshy, so you can only eat rice porridge. You can’t eat hard (things) such as duck, and you don’t dare to eat it.* (P7)

*In the beginning, my appetite was not good, and my taste was wrong. It was because my mouth had a weird taste, so I lost a lot of weight.* (P11)

*The main thing that affected me as a kid was whatever side effects of the pills (androgens and cyclosporine), like growing hair, and then also developing early…You suddenly become a little hairy kid in school, and have a laryngeal knob, and your voice becomes thicker than a boy’s, and also (skin) is very dark, which can get some just strange looks from everyone.* (P14)

*Gingival hyperplasia can be worse if you take too much cyclosporine, and also I’ve been taking all kinds of medications for years, and now my blood sugar and kidney function are compromised.* (P16)

For patients with severe aplastic anemia, ATG/ALG, a potent immunosuppressant, is usually used in clinical treatment. After ATG/ALG infusion, patients may develop a variety of rashes, or recurrent serum sickness reactions, mainly characterized by high fever, joint and muscle soreness, and proteinuria.

*After receiving the ATG infusion for some time, my legs and joints were sore, which was a reaction to serum sickness. I couldn’t lift or move, so I could only lie there.* (P4)

*I’ve been on ATG since August 9th of last year… The main side effect is that the eyes often glow white, blurry, which goes away after a while, and then a little migraine.* (P11)

*(After ATG treatment) Inside my joints, so uncomfortable, I just walk around, occasionally sit down, I can’t sleep, uncomfortable death.* (P9)

For patients undergoing hematopoietic stem cell transplantation, the initial step involves pretreatment with chemotherapy, which can potentially lead to hemorrhagic cystitis. Following transplantation, patients may experience varying degrees of acute and/or chronic GVHD, marked by symptoms such as rash, blisters, dermatitis, jaundice, vomiting, abdominal pain, and diarrhea, among others. Furthermore, post-transplantation, infections and interstitial pneumonia are common complications.

*I was very uncomfortable in the transplant bay, vomiting every day, I vomited 11 times a day, or 10 times, which was really exaggerated… And then I had diarrhea all the time because of intestinal ulcers… I had a lung infection last year and lost a lot of weight, less than 80 pounds, and now I have a second lung infection. This time I was admitted for vomiting and nutritional problems, and then I was diagnosed with cholecystitis.* (P12)

*I lost my hair on the fifth day of chemotherapy in the transplant bay, and my mouth ulcer was pretty bad, my skin was itchy and peeling, and then the bottom (urethral opening) was rotten.* (P13)

#### Changes in body image

Changes in body image refer to the changes in appearance or secondary sexual characteristics of patients with AA after receiving treatment. Some interviewees had facial acne, skin darkening, and other changes in physical appearance, which affected their quality of life to a certain extent.

*Several patients got acne on their faces after transplantation. Anyway, I still have acne on my chin.* (P1)

*People who don’t have platelets in the hematology department are very black. At that time, we were as black as black people, haha.* (P8)

In addition, after treatment, patients developed characteristics of virilization, including menopause, thicker voice, and increased hairs. These characteristics might have produced or aggravated adverse effects, such as facial anxiety, especially in female patients with AA.

*After taking this medicine once, menstruation is gone. After all, it is a male hormone.* (P5)

*My friends feel that my voice is more masculine than theirs, and they said why my hair is more than their boys’.* (P6)

For male patients, fatigue, muscle loss and relaxation also affect their appearance and body image.

*I used to be outgoing and bubbly, but after my illness, I became more repressed and introverted… Because I have no energy (after the disease), and the side effects of drugs lead to all kinds of discomfort, I cannot go out often, there are many taboos in diet, and I am not free in daily life, almost no social interaction. I don’t feel like a young man anymore. I feel like a retired man.* (P16)

*I usually like to play basketball in school, of course, this year has not played… My legs are noticeably thinner, and my heart beats when I walk too fast. If I keep lying down, I’m gonna be a wreck.* (P3)

### Theme 2. Psychological symptoms

#### Mood changes related to the stage of the disease

Mood changes related to the stage of the disease refers to the emotional feelings of patients with AA provoked by the disease, which vary according to the different stages of the disease. In the early stages of diagnosis, patients often go into shock and denial, while some patients express fear and anxiety.

*I’m afraid of misdiagnosis, just don’t misdiagnose.* (P2)

*Gosh, I never dreamed that I would get this disease! (alas)* (P7).

*After the diagnosis at that time, I knew that (the disease) was also very serious, the treatment time was very long, and the effect varied from person to person, and I was actually very afraid and anxious.* (P16)

Considering AA characteristics of a long treatment period and repeated changes in symptom severity, some interviewees gradually succumbed to anxiety, depression, and despair, and even wanted to give up treatment.

*At that time, I was very breakdown in front of my wife and daughter, although as a man.* (P9)

*Sometimes I want my husband to just buy me some medicine for me to take it and die. After so many treatments, it can’t be cured. My teeth will still bleed. It affects my life too much.* (P7)

Most long-term chronically ill patients with AA accepted their condition, made adjustments to live with AA, and maintained a calm state of mind.

*People suddenly fell straight from normal, maybe the inner shock will be a bit bigger. I had this AA when I was 8 years old, and there are usually no problems in my 30s, so I feel okay.* (P1)

*Maybe I was sick when I was young, my mother would say, don’t be too good at school, don’t have pressure, stay optimistic and happy is enough, I was taught this way from a young age.* (P14)

#### Change in self-image

Shifts in self-image entail that patients with AA undergo varying degrees of transformation in their self-perception and role cognition due to the evolving nature of the disease. This transformation can stem from a negative assessment of self, prompted by the weight of the illness, or it can result from a reevaluation of self-image and life aspirations following the experience of a “rebirth” post-illness. These shifts have a diametrically opposite effect on their overall quality of life.

When individuals with AA experience these conditions, it necessitates a blood transfusion, indicated by: HGB < 60 g/L; PLT < 10 × 10^9^/L, or PLT < 20 × 10^9^/L with a notable tendency for bleeding. Component blood transfusions are administered based on their specific condition. While some patients exhibit significant improvement post-transfusion, others require at least one component transfusion every 8 weeks on average, with a transfusion dependence lasting ≥ 4 months, classifying it as transfusion-dependent non-severe AA (TD-NSAA). However, these patients often humorously refer to themselves as “vampires.“

*I mean like a man who eats opium, and also a bit like a vampire…but continuous blood transfusion has a great impact on the quality of life!* (P7)

In addition, due to the loss of ability to work, patients with the disease had to rely on others for financial support. Two interviewees believed that they were useless after developing the illness, which probably increased their self-perceived burden.

*I used to be a pillar of support at home, but now I am not… now I am a patient, a useless person.* (P8)

Some interviewees saw themselves as brand new human beings after a nirvana rebirth, especially patients successfully treated with HSCT. They expressed a strong desire to live and the determination to help others after a near-death experience.

*Now I want to live well, and then drive other people, especially the patients in the Aplastic Anemia Alliance Association of the hospital, when I have the opportunity, I want to show my words and give them a little confidence in living.* (P5)

#### Growth resulting from the disease experience

Growth resulting from the disease experience means that with the support of self-belief and help from family members and social care, patients with AA have a new and positive cognition of their own lives. This helps improve their quality of life to a certain extent.

After a near-death experience, some interviewees expressed strengthened personal resolve gained through self-regulation and had a clear plan for their lives.

*I didn’t think about the goal at all before. It’s a kind of messing around and taking one step at a time. Now there is a clearer and slightly longer-term plan.* (P3)

Some interviewees said that after receiving help from others, they must show gratitude by earnestly doing good in the second “continuation” of their lives.

*To me, transplantation is a continuation of life. I should live vigorously with gratitude every day, and make good use of the new life my sister gave me evenly.* (P3)

*This (HSCT) is really a rebirth for me, I am very grateful to my family, I am also grateful to life, those who survive will be blessed… Now I want to go home early, find an easy job that can support myself, and spend more time with my parents.* (P19)

In addition, some interviewees expressed their motivation to live positively after illness and hoped to repay society in the future.

*I am eager to change the current negative state. I must find a way to make myself active. I hope (in the future) I can contribute to society and others (laughs).* (P6)

### Theme 3. Social burden

#### Decline in career development

A decline in career development for patients with AA encompasses more than just a hiatus in their regular work or studies due to illness. It extends to impacting their social interactions, altering life trajectories, and influencing career aspirations. This experience can lead to feelings of being disconnected from society and a strong desire to resume studies or work to regain a sense of normalcy and progress.

*(Being at home for a long time due to illness) I want to go out, I want to play, I want to go to work.* (P5)

*(After getting sick) I was in a state of suspension.* (P3)

However, due to the decline of physical ability, high risk of infection and bleeding, patients with AA need to change their jobs and adjust their family planning. Some middle-aged and elderly patients perceive themselves as weakened by illness, believing they require prolonged periods of rest at home. Younger individuals often find it impractical to continue in their previous occupations, especially those demanding high levels of activity, travel, high stress levels, exposure to chemicals or ionizing radiation, or necessitating night shifts. As a result, their choice of jobs is very limited.

*My husband and I used to run a restaurant, and after I got sick, the restaurant closed down…When I am cured we may go back to our hometown in the countryside, I will do some simple housework at home, and he will go out to be a chef to earn money.* (P13)

*I used to be a doorman, it’s really hard, but the pay is good, more work more… After I leave the hospital, my work will definitely be affected, I don’t know when this treatment will be finished, I don’t know what to do after, a lot of (work) cannot do.* (P17)

*I like to shoot during the day and edit at night… Because I can’t calm down during the day, and I’m more inspired at night… But I have to change my career in the future. I can’t work so hard anymore, and I don’t want to stay up late.* (P4)

In addition, employers’ indiscriminate discrimination based on employees’ health also greatly increases the difficulty of social reintegration of patients with AA.

*Generally, in a new job, the boss does not accept people like me who have not recovered from illness, and no one wants me…so life is neither quality nor guaranteed, and it depends on parents.* (P1)

*Later, the company representative came to visit me, in fact, implied that I should leave, because I asked for sick leave for a long time, the company has to pay “five insurance and one fund”. I don’t know what I’m gonna do, and I don’t think they’re gonna hire me again.* (P17)

#### Perceived burden to the family

Perceived burden to the family means that the disease not only has a great impact on the life of the patient, but also imposes a heavy burden on the patient’s family, including increased financial stress, strained family relations, and anxiety about the health of the family and future generations.

Patients with AA frequently face challenges in maintaining employment until they achieve full recovery. They rely on consistent care and support from their spouses or family members. Additionally, the treatment for AA can be financially burdensome, particularly for ordinary families. This expense can be catastrophic for those without stable employment, akin to a regression to earlier times of hardship.

*After I got sick, it was very stressful for the family. The flow of money in our lives was cut off. I didn’t go to work and depended on my wife to support me. This brought a great disaster to the family, and the quality of life was lost at all.* (P9)

Some patients spent more time, money, and energy after the illness, but their physical condition failed to improve as expected. The disappointing treatment results not only bring negative emotions to patients, but also affect family members, making family relations delicate and tense, and even quarrel.

*I became irritable after I got sick, and after spending so much money, time was wasted, and my parents also had emotions…like my temper is a little bit worse, my mother said a few words about me and I would say something against her.* (P1).

In addition, some patients frequently asked their children to undergo physical examinations for fear that the next generation may be affected as well whereas some did not consider giving birth at all.

*There must be some worries (about fertility) because after all, I have been sick, I am afraid that it will affect the child… so I often ask my daughter for medical examinations for fear of what happens to my daughter.* (P10)

*I was diagnosed with polycystic ovary syndrome due to long-term medication (androgen), which is not easy to get pregnant, and if I get pregnant, it will not only lead to changes in my condition, but also pose a great health risk to the baby… We don’t want to take the risk.* (P14)

#### Social stigma

Social stigma refers to the social prejudice or discrimination against people with AA on the basis of their illness, probably due to the lack of knowledge about AA. Some interviewees said that they were often the butt of gossip after falling ill, which to some extent triggered or aggravated the stigma against them.

*Especially in places like our rural areas, if you have such a serious illness and go back, there will be many people talking about it. Besides, the old women in the countryside will ‘chewing the tongue (a Chinese slang term that means spreading gossip)’.* (P1)

*There may be (looking at me with strange eyes), they may not say it, but they will think in their hearts or say it behind my back.* (P6)

Some patients were reluctant to go out because of fear of being alienated by others. Some interviewees expressed that their normal social lives had been affected because of the reputational damage of gossip.

*I don’t want to go for a walk. When I go out and see people in the village who donated money to me, I can’t say hello to everyone I see, then it feels weird if I don’t say hello… being so careful every day is affecting my life.* (P4)

*Sometimes they (young patients) don’t dare to order takeout and drink bubble tea at home…they will be seen by these older people in the village and say, Ah, this kid is so seriously ill that he still drinks bubble tea.* (P1)

## Discussion

In this study, most patients with AA experienced declined body function, and even after a series of treatments, many patients with AA still had some special internal symptoms, external image changes, and other side effects. These findings are consistent with the results of a previous study [10]. Li et al. found that children with acute leukemia had 8–12 symptoms simultaneously or successively during chemotherapy, and the number, severity, and distress of the symptoms significantly correlated with the functional status and quality of life of the children [24]. Therefore, medical staff should monitor disease progression in patients with AA, carry out targeted interventions to improve AA symptoms, and implement efficient and practical symptom management models to improve the quality of life of patients [25]. To ensure the effective management of AA and address any distressing symptoms or adverse treatment reactions, nursing staff should employ scientifically validated tools for timely and systematic evaluation [26]. For example, Patient-reported Outcome Questionnaire for Aplastic Anemia and Paroxysmal Nocturnal Hemoglobinuria (PRO-AA/PNH) can be used to assess patients’ quality of life [18]. Alternatively, the newly developed tool Quality of Life Scale for Patients with Aplastic Anemia (QLS-AA) [27], which is more in line with the clinical situation in China, can be adopted clinically. Clinical nursing staff can periodically assess the quality of life of patients with AA at different stages, and provide intervention or support measures according to the evaluation results. For example, clinical nurses and community nurses should closely monitor the condition of the skin in patients with AA, especially in the palms, soles, groin, perineum, perianal, and other areas, during and after ATG or HSCT and promptly treat any skin disorders [28]. For patients with AA with drug-induced gingival hyperplasia, nurses should provide guidance on oral hygiene, healthy eating habits, and regular good grooming habits. They can also encourage dentist visits for a dental examination to check for swollen gums and dental plaque [29]. For patients with AA with treatment-related taste disturbance, nursing staff can suggest some simple clinical interventions, such as eating candy to increase taste, or use plastic or ceramic tableware to alleviate metallic taste to improve taste discomfort in patients with AA [30]. Finally, hospitals should improve out-of-hospital follow-ups, establish a specialist nurse follow-up team, and strengthen online evaluation system. Promoting care continuity with internet nursing services approach is important as well. Such services would allow patients with AA to receive physician feedback on their symptoms during the rehabilitation period through the Internet at any time. In addition, AA support groups or AA social media groups also need to be developed, such as WeChat groups for patients with AA and offline AA mutual assistance groups. The development of educational channels in the form of websites can also enable patients with AA to learn more about health knowledge. This would enhance awareness and self-management of symptoms, reduce the patients’ level of uncertainty over disease progression and help promote their quality of life [31].

In our study, all 19 interviewees had varying degrees of negative emotions and displayed diverse emotional reactions at different stages of the disease. In some cases, multiple negative emotions coexisted, consistent with findings of other studies [32]. Additionally, due to the long wait for treatment, some interviewees still showed poor role recognition, especially patients were dependent on blood transfusions, who visualized themselves as “vampires”. This perception may affect the sense of self-worth of patients with AA and enhance self-perceived burden, further aggravating their negative emotions. A previous study found that more than one-third of elderly patients with hematological malignancies experienced moderate to severe self-perceived burden when starting chemotherapy [33]. If negative emotions are not effectively regulated, patients with AA will eventually develop negative coping styles such as escapism and resignation, which will seriously affect their quality of life. Therefore, nurses should monitor the emotional changes of patients with AA in different treatment stages, and provide targeted comfort, encouragement, and counseling according to the type of negative emotions displayed. In addition, patients should be assisted to actively participate in self-care, respond positively to life’s difficulties, change poor self-perception, and improve their sense of self-worth [34, 35]. In addition, nurses should not only support patients to solve problems themselves, but also promote family involvement in care. By promoting meaningful dialogues between family members, the patient’s self-perceived burden on family patients can be reduced [34]. Besides, medical staff should assess the potential of patients with AA to cope with the disease and guide them through discovery of potential “benefits” of the disease to assist patients to make appropriate psychological adjustment. For example, nursing staff can guide patients with AA to reflect on and set a lower self-evaluation frame of reference for negative events to maximize the positive significance of negative events [36]. Finally, tailoring the re-establishment of life goals to the specific conditions of patients with AA can foster self-realization and imbue life with greater meaning. This approach not only diminishes negative emotions like anxiety and disappointment, but also enhances overall quality of life [37].

In our study, AA had varying degrees of impact on career development, family burden, social prejudice, etc., contributing to a series of family and social problems. The trauma to the patient was more serious and long lasting than the physical threat posed by the disease and treatment. Studies showed that of patients with AA who received HSCT or IST therapy, 74.6% and 81.2% respectively were reintegrated into society [38]. Due to the huge economic expenditure, family relationships of some interviewees were not harmonious, with the occupational interruption after the illness further increasing the financial burden. In America, the mean-adjusted incremental cancer-related costs for commercially insured hematologic malignancy patients was US$ 399,011 in the overall observation period including the pre-diagnostic and pre-HCT periods combined [39]. The corresponding mean-adjusted incremental cancer-related costs for Medicare supplemental patients was US$ 195,575 for the same period. In China, the average family economic burden of children with acute leukemia is more than US$ 73,950, with direct medical costs accounting for 62.59% of total spending, and the average social support provided to children is more than US$ 29,580 [40]. According to the above data, we see that although the current medical reimbursement from the government can help patients with AA solve part of their economic problems, the treatment and care of patients with AA still require high personal or family expenses. Therefore, it is advisable for the government to expedite the enhancement of policies like the medical insurance reimbursement system and charitable relief funds, while also implementing social safety measures. This will help alleviate the financial burden of AA on Chinese families [40]. In addition, medical staff should encourage families of patients with AA to actively improve family functioning, and provide sufficient social support to patients, enhance their medical knowledge to improve their confidence in the treatment and strengthen their psychological flexibility [41]. In addition, government departments can increase employment support for patients with AA by providing legal and regulatory guarantees, increasing employer subsidies, and strengthening vocational rehabilitation services. Specific interventions include providing flexible working hours and tasks and promoting the formation of a social security system based on “employees are protected from dismissal due to sickness” policy [42]. These methods are necessary to protect basic employment rights, reduce employment discrimination against patients with AA, promote their reintegration into society, and reduce the financial burden on families. Furthermore, some interviewees could not go out for fear others’ “gossip”. This suggests that patients with AA are ashamed to share their illness experiences with others and the public lacks basic understanding and cognition of AA. Therefore, we call on roleplayers in society to increase awareness of AA and promote positive attitudes and humanistic care for patients with AA by organizing activities such as AA clubs or Aplastic Anemia Alliances Association. Meanwhile. We need to promote peer support to patients, eliminate social prejudice, reduce stigma towards patients with AA, and finally improve their quality of life [43, 44].

### Limitations

This study has some limitations. First, theme saturation was a relative concept that was only limited to the findings of this study and might change over time. Second, for practical reasons, most of the samples were relatively young and came from just two hospitals in China. It is known that geographical environment, medical conditions, and social background have potential effects on patients’ quality of life. So if there are older people from other parts of China, other themes may be revealed. Third, a spouse attended one of the interviews, which might have affected the participants’ openness and truthfulness. These factors should be considered when interpreting this study’s findings.

## Conclusion

This study contributes new knowledge to the current understanding of quality of life experiences of Chinese patients living with AA. With the diagnosis of AA and side-effects of AA treatments, physical, psychological, and social aspects of the patient’s life are changed significantly. Therefore, health care professionals should endeavor to fully understand the feelings of these patients. They should provide them with effective and individualized medical guidance as well as related information on social support to enhance their confidence and improve their quality of life.

### Electronic supplementary material

Below is the link to the electronic supplementary material.

**Supplementary Material 1**: Figure of themes and subthemes of the study

**Supplementary Material 2**: Tables of demographic and clinical characteristics of the participants and themes and subthemes of the study

**Supplementary Material 3**: Revised manuscript

**Supplementary Material 4**: Comment and reply 1

**Supplementary Material 5**: Consolidated criteria for reporting qualitative studies (COREQ): 32-item checklist

## Data Availability

The datasets generated and analyzed during the current study are not publicly available due to participant privacy concerns but are available from the corresponding author on reasonable request.

## Abbreviations

AA: aplastic anemia
IST: immunosuppressive therapy
HSCT: hematopoietic stem cell transplantation
OS: overall survival
ATG: Anti-thymocyte globulin
ALG: Anti-lymphocyte globulin
HGB: hemoglobin
ANC: absolute neutrophil count
PLT: platelet
GVHD: graft-versus-host disease
