# Supplementary figures and images for: Experience of life quality from patients with aplastic anemia: a descriptive qualitative study
Source: Orphanet J Rare Dis. 2023 Dec 21;18:393. doi: 10.1186/s13023-023-02993-y (PMC10740222; doi:10.1186/s13023-023-02993-y)

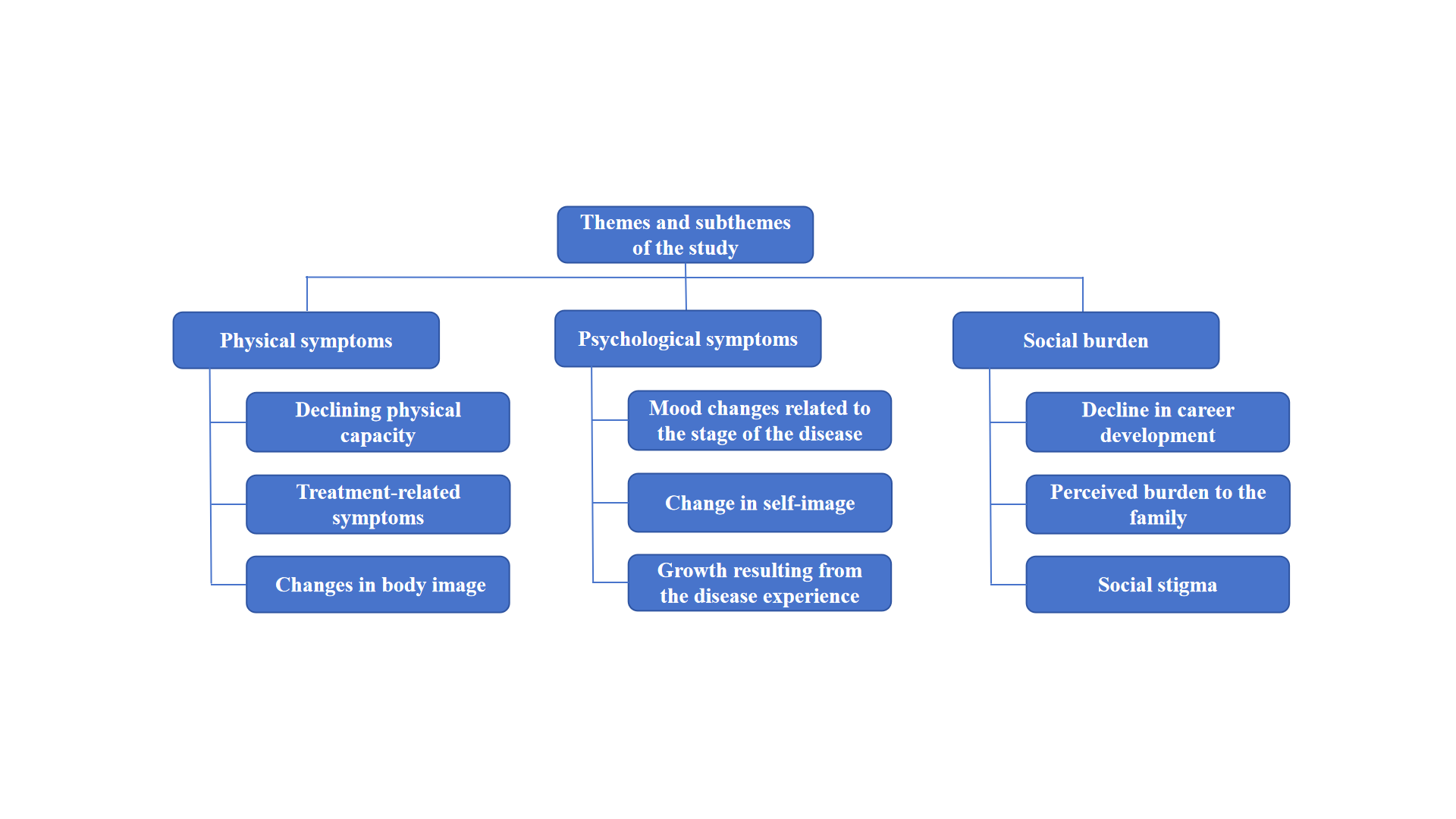

Supplement: Supplementary file 1 — Supplementary Material 1: Figure of themes and subthemes of the study [file 13023_2023_2993_MOESM1_ESM.tif]
