# Supplementary material for: Experience of life quality from patients with aplastic anemia: a descriptive qualitative study
Source: Orphanet J Rare Dis. 2023 Dec 21;18:393. doi: 10.1186/s13023-023-02993-y (PMC10740222; doi:10.1186/s13023-023-02993-y)
Supplement: Supplementary file 2 — Supplementary Material 2: Tables of demographic and clinical characteristics of the participants and themes and subthemes of the study [file 13023_2023_2993_MOESM2_ESM.docx]

**Supplementary Table 1. Demographic and clinical characteristics of the participants.**

| **No.** | **Gender** | **Age**  **(years)** | **Marital status** | **Education level** | **Main caregiver** | **Work/Study situation** | **Economic dependence** | **Course of disease (years)** | **Degree of anemia** | **Blood transfusion situation** | **Treatments since disease** |
| --- | --- | --- | --- | --- | --- | --- | --- | --- | --- | --- | --- |
| 1 | Male | 33 | Single | Secondary | Mother | Unemployed | Total dependence | 25 | Normal | No | 1,3,4,5,6,7 |
| 2 | Female | 41 | Married | Tertiary or above | Husband | Unemployed | Independence | 0.04 | Sever | Yes | 5,6 |
| 3 | Male | 19 | Single | Secondary | Mother | Schooling suspension | Total dependence | 1 | Sever | Yes | 1,3,4,5,7 |
| 4 | Male | 26 | Single | Tertiary or above | Mother | Employed | Partial dependence | 1.25 | Mild | No | 2,3,4,5,6 |
| 5 | Female | 30 | Single | Secondary | Mother | Unemployed | Total dependence | 8 | Normal | No | 1,3,4,6 |
| 6 | Female | 20 | Single | Secondary | None | Attending school | Partial dependence | 2.25 | Moderate | No | 3,4,5,6 |
| 7 | Female | 47 | Married | Primary or less | Husband | Employed | Independence | 0.8 | Mild | Yes | 3,4,5,6 |
| 8 | Male | 67 | Widowed | Primary or less | None | Retired | Partial dependence | 5 | Sever | Yes | 3,4,5,6 |
| 9 | Male | 51 | Married | Secondary | Wife | Unemployed | Total dependence | 0.9 | Normal | No | 1,3,4,5,6,7 |
| 10 | Female | 23 | Married | Secondary | Husband | Unemployed | Total dependence | 2 | Sever | No | 1,3,5 |
| 11 | Female | 21 | Single | Tertiary or above | Mother | Unemployed | Partial dependence | 0.25 | Sever | No | 1,5,6 |
| 12 | Female | 27 | Single | Tertiary or above | Parent | Unemployed | Total dependence | 2.1 | Mild | No | 1,3,4,5,6,7 |
| 13 | Female | 51 | Married | Primary or less | Husband | Unemployed | Total dependence | 2 | Mild | No | 1,2,3,4,5,6,7 |
| 14 | Female | 26 | Single | Tertiary or above | Parent | Employed | Partial dependence | 22 | Sever | Yes | 3,4,5,6,7 |
| 15 | Male | 24 | Single | Secondary | None | Unemployed | Partial dependence | 0.5 | Sever | Yes | 3,4,5,6,7 |
| 16 | Male | 20 | Single | Secondary | Parent | Schooling suspension | Total dependence | 3 | Sever | Yes | 2,3,4,5,6,7 |
| 17 | Male | 33 | Married | Secondary | Wife | Employed | Independence | 16 | Normal | No | 3,6 |
| 18 | Female | 66 | Married | Secondary | Sons and daughters | Retired | Partial dependence | 1.5 | Moderate | Yes | 3,4,5,6 |
| 19 | Female | 27 | Single | Tertiary or above | None | Employed | Partial dependence | 16.6 | Mild | No | 3,4,5,6 |

Note:1. HSCT, hematopoietic stem cell transplantation; 2. ATG/ALG, anti-thymocyte globulin/anti-lymphocyte globulin; 3. CsA, Cyclosporine A; 4.Androgen; 5.Blood transfusion; 6.Traditional Chinese medicine; 7.Eltromborp

**Supplementary Table 2.** **Themes and subthemes of the study.**

| **Themes** | **Subthemes** |
| --- | --- |
| Physical symptoms | 1. Declining physical capacity |
|  | 2. Treatment-related symptoms |
|  | 3. Changes in body image |
| Psychological symptoms | 1. Mood changes related to the stage of the disease |
|  | 2. Change in self-image |
|  | 3. Growth resulting from the disease experience |
| Social burden | 1. Decline in career development |
|  | 1. Perceived burden to the family |
|  | 1. Social stigma |
