# Supplementary material for: Experience of life quality from patients with aplastic anemia: a descriptive qualitative study
Source: Orphanet J Rare Dis. 2023 Dec 21;18:393. doi: 10.1186/s13023-023-02993-y (PMC10740222; doi:10.1186/s13023-023-02993-y)
Supplement: Supplementary file 4 — Supplementary Material 4: Comment and reply 1 [file 13023_2023_2993_MOESM4_ESM.doc]

Orphanet Journal of Rare Diseases

OJRD-D-23-00534

Experience of life quality from patients with aplastic anemia: a descriptive qualitative study

**To Editor：**

Thank you for editing and processing our manuscript. Based on your comments, we have revised and replied as follows:

1. Thanks for your check and reminder, we checked and updated COREQ according to the new content and research team, modified content was highlight marked (COREQ32-item checklist-Marked), as well as provided a new formal version (COREQ32-item checklist-New). Other supplementary materials (tables and diagrams) have been updated since the first revision.

2. We have revised the abstract, with particular emphasis on the main findings of this study, which are currently 286 characters.

**To Reviewer #2：**

Thank you for your comments and review of our manuscript.

**To Reviewer #4:**

Thank you very much for making many specific and wise revisions to our manuscript, including the improvement of the study design, the honorific title for patients, the standardized expression of language, etc. We have learned a lot, and we sincerely express our thanks and respect to you. In addition, regarding this revision, we explain as follows:

1.Thank you for your meticulous revision of our manuscript. We think all your suggestions are reasonable, so we accept all your revisions. The revised contents (marked in red in the manuscript) include but are not limited to: ① the standardized expression of drug name and currency; ②‘AA patients’ were replaced by ‘patients with AA’; ③‘laryngeal knot’ is changed to ‘laryngeal knob’；④ Revision of references; ⑤ Other language or grammar problems.

2. This is a really cute question about "chewing the tongue." If you want to know more, I would really like to have more communication with you through email and other means. In short, "chewing the tongue" is a Chinese slang phrase transliterated "jiao she gen（嚼舌根）" in Chinese, which is similar to "gossiped about" and "bandy about" in English. However, “jiao she gen” usually gossips about others with negative comments and speculation. Therefore, the Chinese character "jiao she gen" carries the potential intention of spreading gossip, probing privacy and denigrating images. In the manuscript (lines 405 and 409), we changed it to "gossip" to facilitate understanding, while in the patient's representative quote, we kept the superficial translation and added an explanation in parentheses. We hope that this will not only provide a better understanding for non-Chinese readers, but also preserve the original expression.

At the same time, we were inspired to translate some languages more appropriately, such as "milk tea" to "bubble tea", so as to promote our cross-cultural understanding of language.

3. Since there is no need to make changes in the article, we hereby reply you:

The refrence [14] *Li P. Health-related quality of life and the correlation analysis with social support in patients with acquired aplastic anemia. Shandong University. 2017.*

This is indeed a doctoral degree thesis from Shandong University.

4. We have added 5 patients from northern China respectively in the methods and results sections of the abstract and the main text, but there is no significant difference in their quality of life in all aspects. Thank you for your advice.
