# Supplementary material for: Experience of life quality from patients with aplastic anemia: a descriptive qualitative study
Source: Orphanet J Rare Dis. 2023 Dec 21;18:393. doi: 10.1186/s13023-023-02993-y (PMC10740222; doi:10.1186/s13023-023-02993-y)
Supplement: Supplementary file 5 — Supplementary Material 5: Consolidated criteria for reporting qualitative studies (COREQ): 32-item checklist [file 13023_2023_2993_MOESM6_ESM.pdf]

Table: Consolidated criteria for reporting qualitative studies (COREQ): 32-item checklist

No	Item	Guide question/description	Answer
	**Domain 1: Research team and reflexivity**		
	Personal characteristics		
1.	Interview/facilitators	Which author/s coordinated the interview or focus groups?	Ting Liu and Pan Yue organized the interviews together.
2.	Credentials	What were the researcher's credentials?	three researcher is a PhD graduate and another is a master's graduate.
3.	Occupation	What was their occupation at the time of the study?	one researcher is a postdoctor, two were clinical nurses and the others are teacher.
4.	Gender	Was the researcher male or female?	one of the researchers was male and five were female
5.	Experience and training	What experience or training did the researcher have?	Train:<br>Read books such as "Qualitative Research in Nursing: Theory and Cases", Qualitative Research Workshop, Seminar on theory and practice of qualitative research, Training in qualitative data collection, Training in Qualitative Data Analysis, Participate in classroom interview practice and data analysis process, Training for Data Management, Communication with qualitative research experts.<br><br>Experience:<br>The team has published 5 articles related to qualitative research.
	Relationship with participants		
6.	Relationship established	Was a relationship established prior to study the commencement?	Yes.
7.	Participant knowledge of the interviewer	What did participants know about the researcher?	Reasons for conducting the study and the institution conducting the researcher.

8.	Interviewer characteristics	What characteristics were reported about the interviewer/facilitator?	Two interviewers are both graduate students, working and practicing in the hematology department. We were interested in the quality of life of patients with aplastic anemia in clinical practice, but few studies have examined the experience of aplastic anemia patients in the quality of life, so we did this study. However, previous internship experience may make researchers have more empathy for patients, which may lead to some preconceived assumptions in the interview.
	**Domain 2: Study design**		
	Theoretical framework		
9.	Methodological orientation and theory	What methodological orientation was stated to underpin the study?	Descriptive qualitative research.
	Participant Selection		
10.	Sampling	How were participants selected?	Purposive sampling.
11.	Method of approach	How were participants approached?	Face-to-face.
12.	Sample size	How many participants were in the study?	There were 19 participants in the study.
13.	Non-participation	How many people refused to participate or dropped out? Reasons?	None.
	Setting		
14.	Setting of data collection	Where was the data collected?	Traditional Chinese Medicine treatment room (4 participants), the ward stem cell collection room (3 participants), the sterile transplantation warehouse (3 participants), the patient single ward (1 participant), the education room (5 participants)
15.	Presence of non-participants	Was anyone else present beside the participants and the researchers?	Yes, a spouse was present at one of the interviews due to the participant’s illness. (P2)
16.	Description of sampling	What are the important characteristics of the sampling?	Age, marital status, education level, main caregiver, work/study situation, economic dependence, course of disease, the degree of anemia, current blood transfusion situation, treatments since disease.

	Data collection		
17.	Interview guide	Where questions, prompts, guides provided by the authors? Was it pilot tested?	Yes, the question guides were designed and pilot tested to participants with similar characteristics of the study in a different setting of the main study area. The final guide was revised, and approved by all authors.
18.	Repeated interviews	Were repeat interviews carried out? If yes, how many?	No.
19.	Audio/visual recording	Did the research use audio or visual recording to collect the data?	Yes, audio recording was used to collect data with all participants.
20.	Field notes	Were field notes made during and/or after interview or focus group?	Yes, field notes were made during interviews.
21.	Duration	What was the duration of the interviews or focus group?	The duration of interview lasted between 31 minutes and 141 minutes.
22.	Data saturation	Was data saturation discussed?	Yes, data saturation was discussed during data collection.
23.	Transcript returned	Were transcripts returned to participants for comments and/or correction?	No. Transcriptions were not returned to each individual participant. But, interviews were held with some participants to discuss the preliminary findings of data analysis.
	**Domain 3: Analysis and findings**		
	Data analysis		
24.	Number of data coders	How many data coders coded the data?	2.
25.	Description of the coding tree	Did authors provide a description of coding tree?	No, we use the traditional manual coding method and gather meaning units according to the original coding to obtain the theme tree. We don't use coding software like Nvivo, so there is no coding tree.

26.	Derivation of themes	Were themes identified in advance or derived from the data?	Themes were derived from the data.
27.	Software	What software, if applicable, was used to manage the data?	None.
28.	Participant checking	Did participants provide feedback on the findings?	Yes, we asked two participants to discuss and feedback our findings.
	Reporting		
29.	Quotations presented	Where participant quotations presented to illustrate the themes/findings? Was each quotation identified?	Yes. Each theme was presented by quotations and each quotation was identified by participant number.
30.	Data and finding consistence	Was there consistency between the data presented and the findings?	Yes.
31.	Clarity of major themes	Were major themes clearly presented in the findings?	Yes.
32.	Clarity of minor themes	Is there a description of diverse cases or discussion of minor themes?	Yes.
